# Performance and Enhancement of Various Fillers Guiding Vanadium (V) Bioremediation

**DOI:** 10.3390/ijerph192214926

**Published:** 2022-11-13

**Authors:** Liting Hao, Lin Li, Bangyan Wang, Xinli Wang, Jinkai Shi, Chen Shi, Xiaodi Hao

**Affiliations:** Sino-Dutch R&D Centre for Future Wastewater Treatment Technologies/Key Laboratory of Urban Stormwater System and Water Environment, Beijing University of Civil Engineering and Architecture, Beijing 100044, China

**Keywords:** vanadium (V), fillers, straw, adsorption, bioremediation

## Abstract

Bioremediation of vanadium (V) pollution in groundwater is an emerging topic. However, knowledge of V in a biogeochemical process is limited and long-term effective removal methods are lacking. V(V) remediation processes by various kinds of auxiliary fillers (maifanite-1, maifanite-2, volcanic rock, green zeolite and ceramsite), agricultural biomass and microbial enhancing were explored in this study. In tests without inocula, the V(V) removal efficiencies of ceramsite (inert filler) and maifanite-2 (active filler) were 84.9% and 60.5%, respectively. When inoculated with anaerobic sludge, 99.9% of V(V) could be removed with the synergistic performance of straw and maifanite-2. TOC (Total Organic Carbon), trace elements and three-dimensional fluorescence analyses confirmed that maifanite-2 was the most suitable among various fillers in biological V(V) removal systems with straw. This study provides a collaborative method (adsorption–biology) by using straw with maifanite-2 in V(V)-contaminated groundwater. The knowledge gained in this study will help develop permeable reactive barrier technology to repair polluted groundwater to put forward a reasonable, effective and sustainable environmental treatment strategy.

## 1. Introduction

In modern industry, vanadium (V) is widely used in various fields, particularly in aerospace, steelmaking, chemical production and batteries [1]. It is estimated that the annual consumption of V will reach 130.1 kilotons by 2024 [2]. V mining, fossil fuel smelting and product manufacturing have caused heavy V pollution [3]. V concentration of the fly ash pits was reported to be as high as 58.6 mg/L [4]. People who took 4.5–18 mg V/d for 6–10 weeks suffered from green tongue, spasms and diarrhea, and even harmed to nerve tissues [5,6]. Therefore, V contamination is in urgent need of treatment.

In recent years, one reliable approach to V pollution has been the adsorption, and researchers have primarily focused on low-cost adsorbents, such as agricultural by-products and industrial wastes [7,8,9]. Corn straw, wheat straw and so on have been widely used as low-cost adsorbents [10,11]. Volcanic rocks have good physical properties such as great porosity and specific surface areas, which make them excellent adsorbents [12]. Zeolite and ceramsite have good adsorption and ion exchange capacities [13].

Furthermore, numerous microbes could reduce V(V) to V(IV) to reduce its toxicity. The toxicity of V increases with its valence and solubility, and V(V) is the most toxic [14]. Heterotrophic microorganisms with organic and inorganic substances as electron donors have attracted the interest of scholars. Organic electron donors such as straw and sawdust and inorganic electron donors such as H_2_, S (0), Fe (0) and FeS were proved to be effective in microbial V(V) reduction [15,16,17,18,19]. The aggregation of heterotrophic bacteria in volcanic pores can adequately degrade and utilize COD, which is conducive to the growth of microorganisms [20]. It has been reported that maifanite can provide trace elements that are a part of the microbial enzyme system. Studies have shown that coenzymes and cofactors of anaerobes are highly dependent on the existence of trace elements (Fe, Mo and Ni) [21]. Adding trace element Fe can improve the enzyme activity of anaerobic digestion, promoting the growth of anaerobic-activated sludge [22]. Moreover, trace elements elevate the enzyme reaction related to the degradation of COD and accelerate the biochemical reaction of microorganisms so as to promote metabolism and enhance the microbial reduction of V(V) [12,23].

There are several methods used for the treatment of V, and each method has several advantages and disadvantages. To find a more efficient, pollution-free and economic way, the adsorptive and biological combined removal using several auxiliary filters (maifanite-1, maifanite-2, volcanic rock, green zeolite and ceramsite) was investigated. Microbial-enhanced adsorptive removal of V(V) was carried out in this work. The best V(V) removal performance was revealed by the analysis of the whole combined process. The effect of various kinds of auxiliary fillers on the V(V) bio-reduction process was explored. The results obtained in this study can contribute to the effective bioremediation of a V(V)-contaminated environment.

## 2. Methods and Materials

### 2.1. Preparation of Materials

Maifanite-1, maifanite-2, black volcanics, green zeolite and ceramsite came from Linyi of Shandong Province, Dengfeng of Henan Province, Zhangjiakou of Hebei Province, Weixian of Shandong Province and Zhengzhou of Henan Province (China), respectively. In total, 1–3 mm straw and 3–5 mm fillers were screened, rinsed with tap water and deionized water (3 times), then put into the 40 °C oven. Serum bottles, rubber stoppers, aluminum caps, beakers, tweezers and volumetric bottles were washed with deionized water. The materials above along with centrifugal tubes and 0.45 μm filter membranes were disinfected by UV (4 h) prior to use.

### 2.2. Experimental Procedure

In order to determine the synergetic effect with fillers, straw addition and microbial addition were considered in the two tests. Batch experimental reactors and the specific experimental settings are shown in Figure S1 and Table 1, respectively. There were 6 groups with a total of 12 serum bottles in Test 1. In total, 250 mL actual groundwater in Beijing with NaVO_3_ (75 mg/L V(V)) and 2.5 g straw were added to each reactor. Then, 10 g prepared fillers (maifanite-1, maifanite-2, black volcanics, green zeolite and ceramsite) were added to reactors, labeled MF-1, MF-2, BV, GZ, CM. The blank group was set up without fillers. All reactors were put in the dark (35 ± 2 °C).

To enhance the V(V) bio-reduction process, sludge was added in Test 2 (3 groups, 6 serum bottles) as inocula. Sludge was taken from the secondary sedimentation tank of Beijing Gaobeidian sewage treatment plant and domesticated (3 months) by refreshing the fresh nutrient solution once every 3 d. Nutrient solution contained the following components (per liter): NH_4_Cl (0.1557 g), CaCl_2_ (0.2464 g), MgCl_2_·6H_2_O (1.0572 g), NaCl (0.4459 g), KCl (0.0283 g), NaHCO_3_ (0.8082 g), KH_2_PO_4_ (0.0299 g), C_6_H_12_O_6_ (0.7500 g) and NaVO_3_ (0.1795 g). Sludge was centrifuged 30 min (3000× *g*) with supernatant skimmed, and the precipitated part was transferred to reactors (maifanite-1, maifanite-2 or black volcanics). Next, 250 mL actual groundwater in Beijing with NaVO_3_ (75 mg/L V(V)), 2.5 g straw and 10 g prepared fillers were also added, the same as Test 1, labeled MF-1-S, MF-2-S, BV-S. The whole experimental period was 168 h with the sampling time every 24 h. After passing through the 0.45 μm membrane, liquid samples were refrigerated at 4 °C for further analysis. The information of chemical reagents and instruments used in this study are shown in the Table S1.

### 2.3. Analysis Methods

Aqueous samples were filtered using 0.45 μm membrane before analysis. V(V) existence was analyzed by spectrophotometry. 2-(5-bromo-2-pyridylazo)-5-diethylaminophenol (5-Br-PADAP) was used as a chelating agent for determining V(V) at 601 nm [18]. The total V and microelement in the solution were determined by ICP-OES (ICAP7200, Thermo Fisher Scientific, Waltham, MA, USA). TOC was analyzed by TOC analyzer (vario TOC cube, DKSH, Zürich, Switzerland). The pH level was monitored by using a pH meter. The three-dimensional fluorescence excitation emission matrix (EEM) quantified the changes in different areas of DOM (dissolved organic matter). The total microorganisms’ count was determined using 3 M Petrifilm™ (6406, Saint Paul, MN, USA).

The V(V) removal efficiency (*RE*) was calculated by Equation (1).

(1) $$RE=\frac{C_{0}-C_{e}}{C_{0}}\times 100\%$$

*C*_0_: the initial concentration (mg/L); *C_e_*: the final concentration (mg/L).

## 3. Results and Discussion

### 3.1. Effect of Different Fillers on V(V) Removal Process

**Figure 1:**
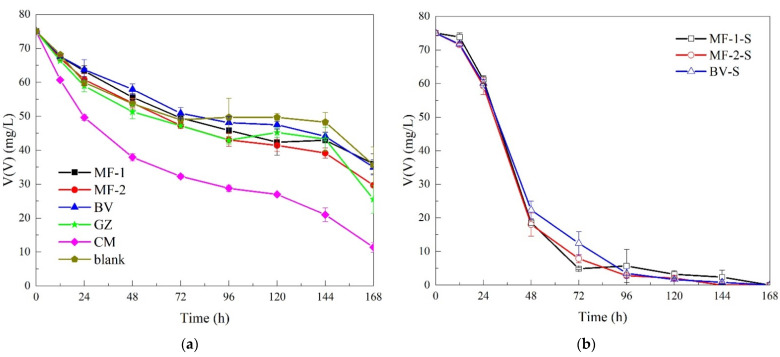
(**a**) V(V) removal by straw and various fillers (Group MF-1, MF-2, BV, GZ, CM and blank); (**b**) enhanced by additional inocula (Group MF-1-S, MF-2-S and BV-S).

**Figure 2:**
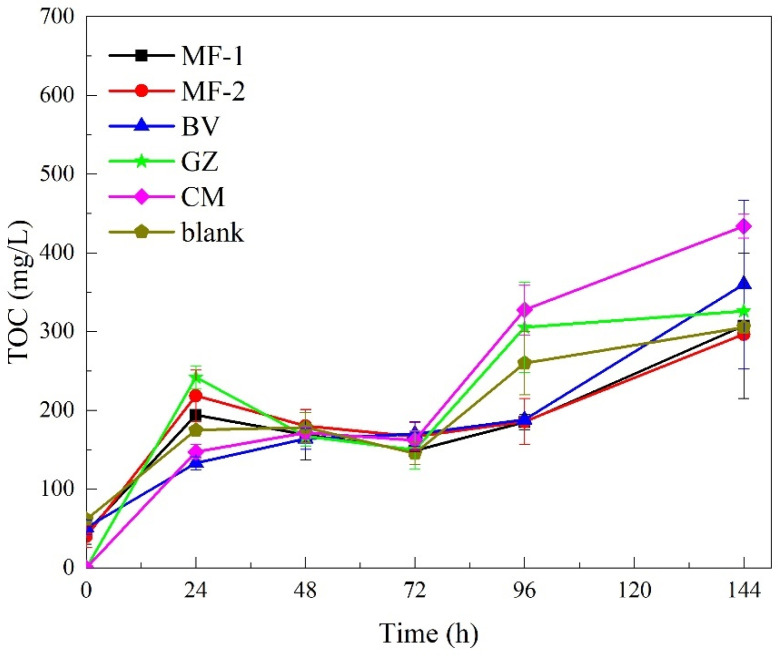
TOC during V(V) removal processes by straw and various fillers (Group MF-1, MF-2, BV, GZ, CM and blank).

In the tests without inocula, V(V) decreased continuously in all groups. It was the most obvious in Group CM (ceramsite), and V(V) removal was the fastest from beginning to end, with its efficiency as high as 84.9% in 168 h. V(V) removal efficiency was 66.0% in Group GZ (green zeolite). The removal efficiency of V(V) in Group MF-2 (maifanite-2) was about 60.5% (168 h), and that in Group MF-1 (maifanite-1) and Group BV (black volcanics) were about 52.1% and 53.4% (Figure 1a). TOC in each group was stable for a short time after the rapid accumulation on the first day and then decreased slightly, then gradually increased after 72 h (Figure 2). This might be owing to the straw continuing to dissolve out of carbon and into the liquid. TOC increased at the beginning. Subsequently, microbial growth and V(V) reduction by using carbon might be faster than that of straw dissolution. TOC concentration in the liquid samples decreased slightly. Then, microbes were resistant to the toxicity of the external environment, and probably did not urgently reduce the toxicity. Thus, the amount of carbon dissolved was greater than that utilized by microorganisms at this stage. TOC in almost all groups was higher than that in the blank group at 144 h. This might be owing to auxiliary fillers enhancing carbon dissolution. In the Group CM, TOC at the endpoint was 434.1 mg/L. The status of the higher TOC concentration in liquid samples (Group CM) might be reflected by the large number of organic substrates dissolving from straw with fewer being consumed microbiologically.

From EEM results, it could be seen that the soluble microbial products (IV zone) of all kinds of fillers showed an upward trend (Table 2). It showed that there were indigenous microorganisms in the straw. However, the humus-like substance (V zone) showed a certain difference. It increased slowly in the reactors with maifanite and black volcanics (Group MF-1, MF-2, BV), while it clearly decreased with green zeolite and ceramsite (Group GZ, CM). Maifanite and black volcanics as active fillers cooperated with in situ microorganisms to hydrolyze straw, and the strength of humus-like substances (V zone) increased. Similar reports were reported in previous studies, such as the strong affinity and adhesion of black volcanics to biofilms [20]. Heterotrophic bacteria were proven to make fuller use of substrates to complete the enrichment process in volcanic pores [12]. In comparison, green zeolite and ceramsite had good adsorption properties as inert fillers. V(V) pollutants in the solution and humus-like substances could be adsorbed, so the strength of the V zone decreased slowly.

### 3.2. Synergism between Various Fillers and Microbes

Among the three kinds of fillers inoculated with domesticated sludge, V(V) removal in Group MF-2-S was the most significant (>99.9%, 144 h), while those in Group MF-1-S and Group BV-S reached more than 99.9% by 168 h (Figure 1b). Tests with operation conditions and performance results in this study and previous studies are shown in Table S2. The 3 M test results showed that different fillers combined with a solid carbon source had a significant enrichment effect on microbes (from 10^5^ to 10^6^). At the same time, the filler had a more pronounced enrichment effect on the primordial microbes. Compared with the original microbes, domesticated microbes need adapt to the conditions of using macromolecular carbon sources (lignin, cellulose and so on). The number of colonies in Groups MF-1-S and MF-2-S with maifanite were consistent with better removal of V(V) performance, especially with maifanite-2 (Table 3). The micropore sponge structure of maifanite could increase the activity of microorganisms [24,25]; moreover, its amendment was adequate to buffer the pH to improve the microbial living environment [26]. These two reasons might further enhance the V(V) bio-reduction. In addition, maifanite could promote the degradation of organic matter and enhance the immobilization of heavy metals [26]. Furthermore, the dissolution of elements such as calcium and zinc from maifanite could be helpful for mineralization [27]. Oxides and calcium carbonates might cause precipitation reactions to fix heavy metals [28].

Three-dimensional fluorescence detection showed that maifanite and black volcanics significantly enhanced the microbial activity in reactors with sludge. It was shown that the addition of maifanite could increase the humic-like substances [29], the main substance produced by hydrolysis and the dissolution of straw. Humus could promote electron transfer in the system [30]. For example, dissolved humus used as an electron shuttle promoted the reduction of Fe (III) in the sediment [31,32]. Humus transferred electrons from the bacteria to the oxide surface and enhanced extracellular electron conduction, significantly accelerating the microbial reduction of iron oxide and metal ions [33,34]. Meanwhile, it was used as a recyclable electron shuttle to accelerate the redox process in the redox treatment of pollutants [30]. Hydroxyl in humus could provide electrons with the ability to reduce heavy metals, and the reaction rate is closely related to the pH of the reaction system [35]. In acidic conditions, the reduced V(IV) bound to oxygen-supplying atoms on the carboxylic acid site of humic acid [36]. V(IV) formed a stable complex with humic acid [37]. It was speculated that humus-like substances accelerated the electron transfer in the process of V(V) reduction and helped to maintain the stable state after V(V) reduction.

The intensities (I and II regions) of all groups constantly changed during the test, which may come from the hydrolysis of straw and the degradation of substances in the V region [38,39]. The intensity of soluble microbial products (IV region) in the early stage (0 h) of Groups MF-1-S, MF-2-S and BV-S was more obvious (Table 2). The soluble microbial by-product in the IV region was related to cellular substances and their secretions and showed the microbial activity [40]. Tryptophan-like substances in dissolved organic matter can bind to heavy metals [41]. Therefore, it was speculated that tryptophan produced in the biological system complexed with V(V), which may be the potential mechanism of V(V) removal in this study. In the meantime, soluble microbial products contained hydroxyl, carboxyl and other functional groups helpful for microbial V(V) bio-reduction [42].

**Figure 3:**
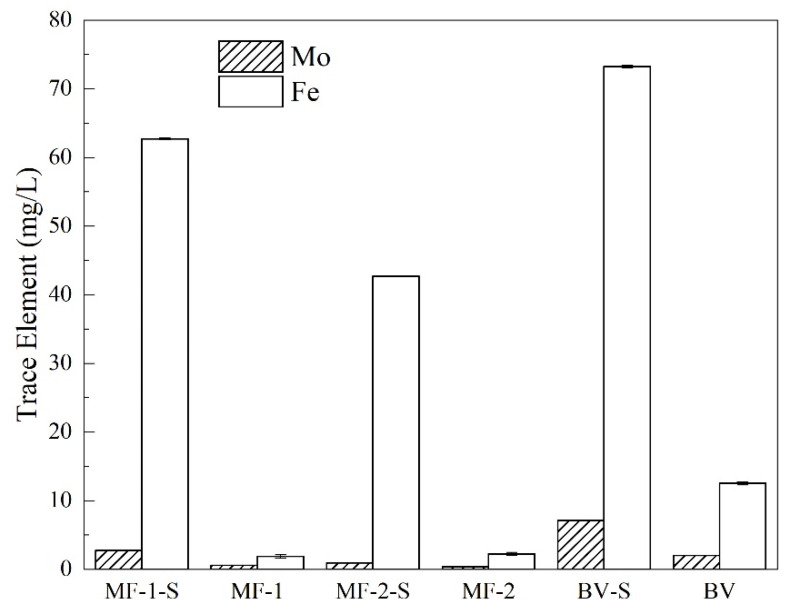
Trace elements’ concentration (Mo and Fe) in Groups MF-1, MF-1-S, MF-2, MF-2-S, BV and BV-S.

In addition to carbon, trace elements played a crucial role in the growth and metabolism of anaerobic microorganisms [43]. Trace elements were commonly parts of the cofactors in the enzyme system, and were vital to the enzyme system [21]. The coenzymes and cofactors of anaerobes were highly dependent on the existence of these metals (Ni, Fe and Mo) [21]. Trace elements often existed in some reductase, such as F420-reductase containing Fe and Ni in the reductive pathway from CO_2_ to CH_4_ [44]. In this study, the trace elements in the initial V contamination solution were detected. Fe and Mo were below the detection limit in the initial solution. Mo and Fe in the liquid samples were also determined at the end of the experiment. A higher Fe concentration (42–73 mg/L) was detected in all the experimental groups inoculated with sludge and the Mo concentration was 0.4–7.1 mg/L (Figure 3). The addition of inocula accelerated the dissolution of trace element Fe from maifanite. Fe was reported to improve the microbial enzyme activity of the anaerobic digestion of agricultural and forestry wastes [45]. It was speculated that the presence of microorganisms promoted the dissolution process of trace elements. It was also reported that maifanite could extract cations such as Fe and Mg [46]. Biofilm could use trace elements (such as Fe) as growth sources [46]. In the dissolution tests, all the experimental groups (MF-1-S, MF-2-S and BV-S) had a certain amount of Mo. Mo was an element in the cofactor of nitrate reductase [47].

Trace elements played an essential role in improving the stable operation of the anaerobic reactor [22]. The addition of trace elements such as Fe, Mo and Ni can accelerate the transformation rate of the substrate [23], thus enhancing the reduction efficiency of V(V). The detection of trace elements in this experiment showed that maifanite and black volcanics can continuously dissolve a certain amount of trace elements with microbes in reactors. The dissolution of Fe and Mo from black volcanics was more than that from maifanite. However, in the macro reaction system, trace elements were accompanied by the dynamic process of dissolution–consumption. To some extent, it might be reflected in the low utilization efficiency in the black volcanics group. It may be one of the reasons that its V(V) removal performance was worse than that with maifanite.

### 3.3. Geochemical and Environmental Implications

Maifanite and black volcanics used in this study are widely distributed and are low-cost materials. The physical and chemical properties of the above materials produced by various producing areas and mining methods are different. Active auxiliary fillers combined with straw have been shown to enable VRB (Vanadium Reducing Bacteria) in the reactors to make good use of the dissolved substrate and trace elements as nutrients. In the early stages, there was a certain intensity about the soluble microbial products (IV zone). VRB quickly adapted to the high V(V) concentration environment in inocula and removed V(V) pollutants rapidly and stably. In addition, the adsorption of straw and fillers, and the participation by in situ microbes cannot be ignored. Even with the lack of enhanced VRB, V(V) removal efficiency still reached more than 50%. This was also shown by the 3 M results. Although the high concentration of V(V) was obviously toxic to organisms, it had an obvious enrichment effect for microbes with the supplement of straw and fillers. To some extent, Group MF-2-S, with the best enrichment effect, showed the best V(V) remediation performance. Thus, V(V) bio-reduction was enhanced by auxiliary fillers, and maifanite-2 was the most suitable filler for the straw biological V(V) removal system in the study. It has significant implications for the remediation of V(V) contaminated groundwater.

## 4. Conclusions

Based on the current concept of treating pollution by using waste material, this study further studied the effect of auxiliary fillers (active fillers and inert fillers) along with agricultural wastes on V pollution. The effect of additional fillers was explored on the microbial V(V) removal system using straw as a carbon source. Inert fillers such as ceramsite had better V(V) adsorption performance (>84.9%). Fillers also showed significant adsorption to various complex carbon sources dissolved from straw. Group MF-2-S (maifanite-2 + straw + sludge) achieved the best V(V) removal performance (>99.9%, 144 h) in the study by the collaborative method (adsorption–biology). The results confirmed that maifanite and other active fillers could enrich a large number of microbes coordinating with straw, and could continuously dissolve a certain amount of trace elements (Mo and Fe) during inocula-enhanced processes. This study improves the efficient, low-cost and long-term technical support for the treatment of V contamination in groundwater.

## Figures and Tables

**Table 1:** Experimental settings.

	Group	Straw (g)	Filler (10 g)	Sludge (mL)	Initial V(V) Concentration (mg/L)
Test 1	MF-1	2.5	Maifanite-1	0	75
	MF-2	2.5	Maifanite-2	0	75
	BV	2.5	Black volcanics	0	75
	GZ	2.5	Green zeolite	0	75
	CM	2.5	Ceramsite	0	75
	blank	2.5	blank	0	75
Test 2	MF-1-S	2.5	Maifanite-1	12.5	75
	MF-2-S	2.5	Maifanite-2	12.5	75
	BV-S	2.5	Black volcanics	12.5	75

**Table 2:** Three-dimensional fluorescence graph at 0, 72 and 168 h.

	Time	0 h	72 h	168 h
Group				
MF-1-S		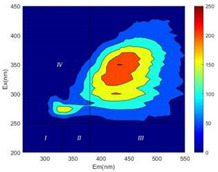	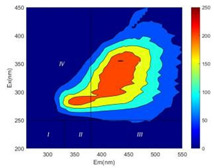	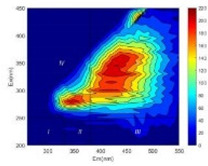
MF-1		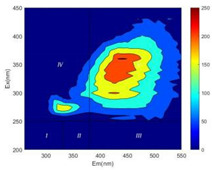	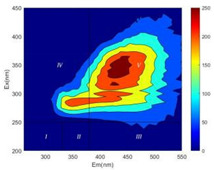	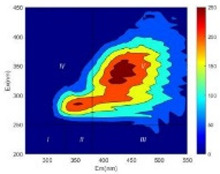
MF-2-S		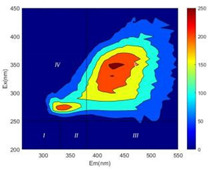	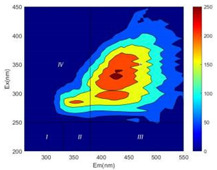	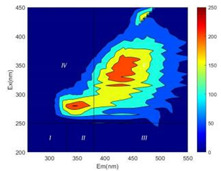
MF-2		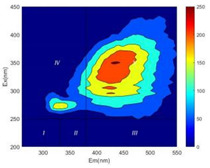	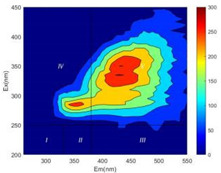	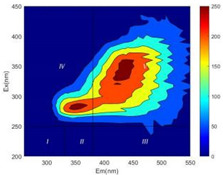
BV-S		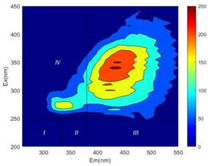	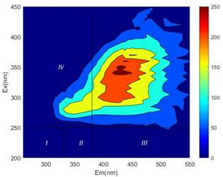	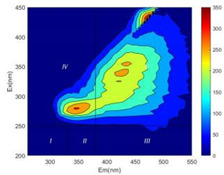
BV		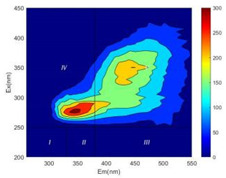	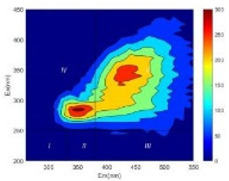	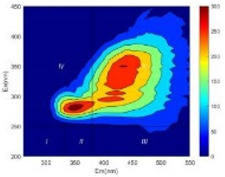
GZ		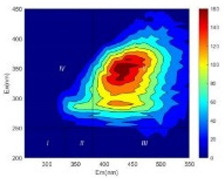	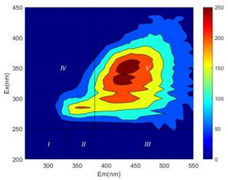	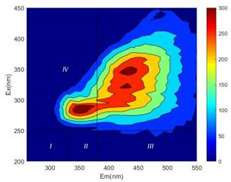
CM		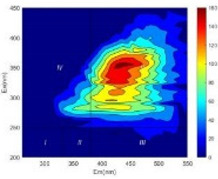	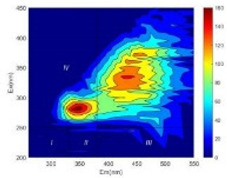	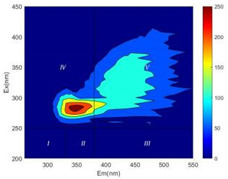
Blank		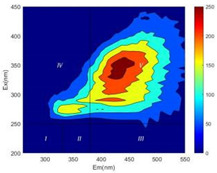	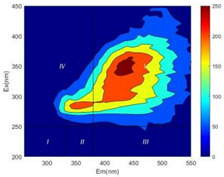	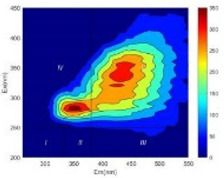

**Table 3:** Total number of bacteria analyzed by 3M.

Group	0 h (×10^5^)	168 h (×10^6^)
MF-1-S	1.5	6.7
MF-1	0	8.7
MF-2-S	1.5	8.6
MF-1	0	8.9
BV-S	1.5	6.1
BV	0	5.7

## Data Availability

The data presented in this study are available on request from the corresponding author. The data are not publicly available due to privacy.

## Supplementary Materials

The following supporting information can be downloaded at: https://www.mdpi.com/article/10.3390/ijerph192214926/s1. Table S1: (a): Information of main chemical reagents used in the study; (b): The main instruments used in the study; Table S2: Tests with operation conditions and performance results in this study and previous studies; Figure S1: The experimental reactors. References [48,49,50,51,52] are cited in supplementary materials.

## References

[1] Imtiaz M., Rizwan M.S., Xiong S., Li H., Ashraf M., Shahzad S.M., Shahzad M., Rizwan M., Tu S. (2015). Vanadium, recent advancements and research prospects: A review. Environ. Int..

[2] Lee J., Kim E., Chung K.W., Kim R., Jeon H.S. (2021). A review on the metallurgical recycling of vanadium from slags: Towards a sustainable vanadium production. J. Mater. Res. Technol..

[3] Burke I.T., Peacock C.L., Lockwood C.L., Stewart D.I., Mortimer R.J., Ward M.B., Renforth P., Gruiz K., Mayes W.M. (2013). Behavior of aluminum, arsenic, and vanadium during the neutralization of red mud leachate by HCl, gypsum, or seawater. Environ. Sci. Technol..

[4] Jia L., Anthony E.J., Charland J.P. (2002). Investigation of vanadium compounds in ashes from a CFBC firing 100 petroleum coke. Energy Fuels.

[5] Anke M. (2004). Vanadium-an element both essential and toxic to plants, animals and humans. Anal. Real Acad. Nac. Farm..

[6] Cooper R.G. (2007). Vanadium pentoxide inhalation. Indian J. Occup. Environ. Med..

[7] Gogoi H., Zhang R., Matusik J., Leiviskä T., Rämö J., Tanskanen J. (2021). Vanadium removal by cationized sawdust produced through iodomethane quaternization of triethanolamine grafted raw material. Chemosphere.

[8] He W., Liao W., Yang J., Jeyakumar P., Anderson C. (2020). Removal of vanadium from aquatic environment using phosphoric acid modified rice straw. Bioremediat. J..

[9] Zhang R., Leiviskä T., Tanskanen J., Gao B., Yue Q. (2019). Utilization of ferric groundwater treatment residuals for inorganic-organic hybrid biosorbent preparation and its use for vanadium removal. Chem. Eng. J..

[10] Hu Y., Liu T., Chen N., Feng C., Lu W., Guo H. (2022). Simultaneous bio-reduction of nitrate and Cr(VI) by mechanical milling activated corn straw. J. Hazard. Mater..

[11] Hao L., He Y., Shi C., Hao X. (2021). Performance and mechanisms for V(V) bio-reduction by straw: Key influencing factors. RSC Adv..

[12] Deng J., Zhou R., Shan X., Ruan W. (2021). Biomass Substrate Carrying Bacteria for Treatment of Micro-polluted River Water. China Water Wastewater.

[13] Wu H., Fan J., Zhang J., Ngo H.H., Guo W., Liang S., Lv J., Lu S., Wu W., Wu S. (2016). Intensified organics and nitrogen removal in the intermittent-aerated constructed wetland using a novel sludge-ceramsite as substrate. Bioresour. Technol..

[14] Yang J., Wang M., Jia Y., Gou M., Zeyer J. (2017). Toxicity of vanadium in soil on soybean at different growth stages. Environ. Pollut..

[15] Hao L., He Y., Shi C., Hao X. (2021). Biologically removing vanadium(V) from groundwater by agricultural biomass. J. Environ. Manag..

[16] Hao L., Liu Y., Chen N., Hao X., Zhang B., Feng C. (2021). Microbial removal of vanadium(V) from groundwater by sawdust used as a sole carbon source. Sci. Total Environ..

[17] Xu X., Xia S., Zhou L., Zhang Z., Rittmann B.E. (2015). Bioreduction of vanadium(V) in groundwater by autohydrogentrophic bacteria: Mechanisms and microorganisms. J. Environ. Sci..

[18] Zhang B., Qiu R., Lu L., Chen X., He C., Lu J., Ren Z. (2018). Autotrophic vanadium(V) bioreduction in groundwater by elemental sulfur and zerovalent iron. Environ. Sci. Technol..

[19] He C., Zhang B., Lu J., Qiu R. (2021). A newly discovered function of nitrate reductase in chemoautotrophic vanadate transformation by natural mackinawite in aquifer. Water Res..

[20] Hao X., Wei L., Qiu F. (2009). Experimental study on enhancing nitrogen removal by effluent recirculation in a BAF reactor with crushed lava packed as carriers. Chin. J. Environ. Eng..

[21] Zandvoort M.H., Hullebusch E.D., Fermoso F.G., Lens P.N.L. (2006). Trace metals in anaerobic granular sludge reactors: Bioavailability and dosing strategies. Eng. Life Sci..

[22] Zhang W., Wu S., Guo J., Zhou J., Dong R. (2015). Performance and kinetic evaluation of semi-continuously fed anaerobic digesters treating food waste: Role of trace elements. Bioresour. Technol..

[23] Cai Y., Hua B., Gao L., Hu Y., Yuan X., Cui Z., Zhu W., Wang X. (2017). Effects of adding trace elements on rice straw anaerobic mono-digestion: Focus on changes in microbial communities using high-throughput sequencing. Bioresour. Technol..

[24] Awasthi M.K., Wang Q., Awasthi S.K., Wang M., Chen H., Ren X., Zhao J., Zhang Z. (2018). Influence of medical stone amendment on gaseous emissions, microbial biomass and abundance of ammonia oxidizing bacteria genes during biosolids composting. Bioresour. Technol..

[25] Yan X., Wang B., Zhang J. (2015). Liquefaction of cotton seed in sub-critical water/ethanol with modified medical stone for bio-oil. Bioresour. Technol..

[26] Wang Q., Wang Z., Awasthi M.K., Jiang Y., Li R., Ren X., Zhao J., Shen F., Wang M., Zhang Z. (2016). Evaluation of medical stone amendment for the reduction of nitrogen loss and bioavailability of heavy metals during pig manure composting. Bioresour. Technol..

[27] Li R., Liu J., Li Y., Cao S., Chen G. (2007). Review on the Application Effects of ’Medica’ Healthy-stone in Hunan Province. Hunan Agric. Sci..

[28] Giagnoni L., Borges L.G., Giongo A., Silveira A., Ardissone A.N., Triplett E.W., Mench M., Renella G. (2020). Dolomite and compost amendments enhance cu phytostabilization and increase microbiota of the leachates from a cu-contaminated soil. Agronomy.

[29] Wang Q., Awasthi M.K., Zhao J., Ren X., Wang M., Li R., Wang Z., Zhang Z. (2018). Utilization of medical stone to improve the composition and quality of dissolved organic matter in composted pig manure. J. Clean. Prod..

[30] Yang Z., Du M., Jiang J. (2016). Reducing capacities and redox potentials of humic substances extracted from sewage sludge. Chemosphere.

[31] Lovley D.R., Coates J.D., Blunt-Harris E.L., Phillips E.J., Woodward J.C. (1996). Humic substances as electron acceptors for microbial respiration. Nature.

[32] Nevin K.P., Lovley D.R. (2000). Potential for nonenzymatic reduction of Fe(III) via electron shuttling in subsurface sediments. Environ. Sci. Technol..

[33] Roden E.E., Kappler A., Bauer I., Jiang J., Paul A., Stoesser R. (2010). Extracellular electron transfer through microbial reduction of solid-phase humic substances. Nat. Geosci..

[34] Zhou S., Chen S., Yuan Y., Lu Q. (2015). Influence of humic acid complexation with metal ions on extracellular electron transfer activity. Sci. Rep..

[35] Wittbrodt P.R., Palmer C.D. (1996). Effect of temperature, ionic strength, background electrolytes, and Fe(III) on the reduction of hexavalent chromium by soil humic substances. Environ. Sci. Technol..

[36] Lu X., Johnson W.D., Hook J. (1998). Reaction of vanadate with aquatic humic substances: An ESR and 51V NMR study. Environ. Sci. Technol..

[37] Ortiz-Bernad I., Anderson R.T., Vrionis H.A., Lovley D.R. (2004). Vanadium respiration by Geobacter metallireducens: Novel strategy for in situ removal of vanadium from groundwater. Appl. Environ. Microbiol..

[38] Luo K., Yang Q., Li X., Chen H., Liu X., Yang G., Zeng G. (2013). Novel insights into enzymatic-enhanced anaerobic digestion of waste activated sludge by three-dimensional excitation and emission matrix fluorescence spectroscopy. Chemosphere.

[39] Wang Y., Zhang J., Sun Y., Yu J., Zheng Z., Li S., Cui Z., Hao J., Li G. (2020). Effects of intermittent mixing mode on solid state anaerobic digestion of agricultural wastes. Chemosphere.

[40] Bridgeman J., Baker A., Marquet C.C., Carstea E. (2013). Determination of changes in wastewater quality through a treatment works using fluorescence spectroscopy. Environ. Technol..

[41] Wu J., Zhang H., He P., Shao L. (2011). Insight into the heavy metal binding potential of dissolved organic matter in MSW leachate using EEM quenching combined with PARAFAC analysis. Water Res..

[42] Liu W., Cheng S., Yin L., Sun Y., Yu L. (2018). Influence of soluble microbial products on the long-term stability of air cathodes in microbial fuel cells. Electrochim. Acta.

[43] Takashima M., Speece R.E. (1989). Mineral nutrient requirements for high-rate methane fermentation of acetate at low SRT. Res. J. Water Pollut. Control Fed..

[44] Michel R., Massanz C., Kostka S., Richter M., Fiebig K. (1995). Biochemical Characterization of the 8-hydroxy-5-deazaflavin-reactive Hydrogenase from Methanosarcina barkeri Fusaro. Eur. J. Biochem..

[45] Khatri S., Wu S., Kizito S., Zhang W., Li J., Dong R. (2015). Synergistic effect of alkaline pretreatment and Fe dosing on batch anaerobic digestion of maize straw. Appl. Energy.

[46] Tsai W., Su T., Hsu H., Lin K., Lin C., Tai T. (2007). Preparation of mesoporous solids by acid treatment of a porphyritic andesite (wheat–rice–stone). Microporous Mesoporous Mater..

[47] Jacques J.G., Burlat B., Arnoux P., Sabaty M., Guigliarelli B., Léger C., Pignol D., Fourmond V. (2014). Kinetics of substrate inhibition of periplasmic nitrate reductase. Biochim. Biophys. Acta-Bioenerg..

[48] Zhang B., Li Y., Fei Y., Cheng Y. (2021). Novel pathway for vanadium (V) bio-detoxification by gram-positive *Lactococcus raffinolactis*. Environ. Sci. Technol..

[49] Wang G., Zhang B., Li S., Yang M., Yin C. (2017). Simultaneous microbial reduction of vanadium (V) and chromium (VI) by Shewanella loihica PV-4. Bioresour. Technol..

[50] Shi C., Cui Y., Lu J., Zhang B. (2020). Sulfur-based autotrophic biosystem for efficient vanadium (V) and chromium (VI) reductions in groundwater. Chem. Eng. J..

[51] Ali M.M.S., Imam D.M., El-Nadi Y.A. (2021). Vanadium (V) removal and recovery by adsorption onto modified activated carbon derived from natural hydroxyapatite. J. Iran. Chem. Soc..

[52] He Q., Si S., Zhao J., Yan H., Sun B., Cai Q., Yu Y. (2018). Removal of vanadium from vanadium-containing wastewater by amino modified municipal sludge derived ceramic. Saudi J. Biol. Sci..

