# Supplementary material for: Performance and Enhancement of Various Fillers Guiding Vanadium (V) Bioremediation
Source: Int J Environ Res Public Health. 2022 Nov 13;19(22):14926. doi: 10.3390/ijerph192214926 (PMC9691244; doi:10.3390/ijerph192214926)
Supplement: Supplementary file 1 [file ijerph-19-14926-s001.zip › ijerph-2002623-supplementary.pdf]

Supporting Information

Performance and Enhancement of Various Fillers Guiding Vanadium (V) Bioremediation

Liting Hao*, Lin Li, Bangyan Wang, Xinli Wang, Jinkai Shi, Chen Shi, Xiaodi Hao*

Sino-Dutch R&D Centre for Future Wastewater Treatment Technologies/Key Laboratory of Urban Stormwater System and Water Environment, Beijing University of Civil Engineering and Architecture, Beijing 100044, China

*Corresponding author: Liting Hao; Xiaodi Hao

E-mail: haoliting@bucea.edu.cn; xdhao@hotmail.com

This supporting information contains:

number of pages: 5

number of tables: 2

number of figures: 1

Table S1. (a) Information of main chemical reagents used in the study.

Laboratory reagents	Manufacturer
Ethanol	Beijing Chemical Pharmaceutical Co., Ltd.
Citric acid	Shanghai Aladdin Biochemical Technology Co., Ltd.
2-(5-bromo-2-pyridylazo)-5-diethylaminophenol (5-Br-PADAP)	Shanghai Aladdin Biochemical Technology Co., Ltd.
$NaHCO_3$	China National Pharmaceutical Group Corporation
$NaVO_3$	Shanghai Aladdin Biochemical Technology Co., Ltd.
HNO_3	Beijing Chemical Pharmaceutical Co., Ltd.
KH_2PO_4	China National Pharmaceutical Group Corporation
H_2SO_4	Beijing Chemical Pharmaceutical Co., Ltd.
NaCl	Beijing Chemical Pharmaceutical Co., Ltd.
$CaCl_2$	China National Pharmaceutical Group Corporation
NaOH	Beijing Chemical Reagent Factory
KCl	China National Pharmaceutical Group Corporation
KBr	Shanghai Aladdin Biochemical Technology Co., Ltd.
Na_2HPO_4	Shanghai Aladdin Biochemical Technology Co., Ltd.
NH_4Cl	China National Pharmaceutical Group Corporation
$C_6H_{12}O_6$	Shanghai Aladdin Biochemical Technology Co., Ltd.
$MgCl_2{\cdot}6H_2O$	China National Pharmaceutical Group Corporation

(b) The main instruments used in the study.

Instrument	Model	Manufacturer
Spectrophotometer	Cary 5000	Agilent Techonlogies
TOC analyzer	vario TOC cube	DKSH, Switzerland
Three-dimensional fluorescence excitation emission matrix	Lumina	Thermo
Ultra-pure water machine	arium comfort	Wiggens
ICP-OES	ICAP7200	Thermo Fisher Scientific
Constant temperature incubator	Nicolet Is5	Thermo Scientific
Mixer	Magnetic Stirrer WH-610D	Wiggens

Table S2. Tests with operation conditions and performance results in this study and previous studies.

Materials	Carbon source	Microorganism	The initial V concentration	Time	Removal Efficiency	Reference
N/A	Citrate (300 mg/L)	*Lactococcus raffinolactis*	50 mg/L	10 d	86.5±2.17%	Zhang et al., 2021
N/A	Citrate (6 mM)	*Shewanella loihica* PV-4	50.6 mg/L	27 d	71.3%	Wang et al., 2017
N/A	N/A	anaerobic sludge	10 mg/L	165 d	99.2±0.6%	Shi et al., 2020
Hydroxyapatite (0.2 g)	N/A	N/A	50 mg/L	30 min	85%	Ali et al., 2021
Amino modified municipal sludge derived ceramic (800 g)	N/A	N/A	50 mg/L	8 h	99.9%	He et al., 2018
Maifanite-1 (10 g)	staw (2.5 g)	anaerobic sludge	75 mg/L	168 h	>99.9%	In this study
Maifanite-1 (10 g)	staw (2.5 g)	N/A	75 mg/L	168 h	52.1%	In this study
Maifanite-2 (10 g)	staw (2.5 g)	anaerobic sludge	75 mg/L	168 h	>99.9%	In this study
Maifanite-2 (10 g)	staw (2.5 g)	N/A	75 mg/L	168 h	60.5%	In this study
Black volcanics (10 g)	staw (2.5 g)	anaerobic sludge	75 mg/L	168 h	>99.9%	In this study
Black volcanics (10 g)	staw (2.5 g)	N/A	75 mg/L	168 h	53.4%	In this study
Green zeolite (10 g)	staw (2.5 g)	N/A	75 mg/L	168 h	66.0%	In this study
Ceramsite (10 g)	staw (2.5 g)	N/A	75 mg/L	168 h	84.8%	In this study
N/A	staw (2.5 g)	N/A	75 mg/L	168 h	52.4%	In this study

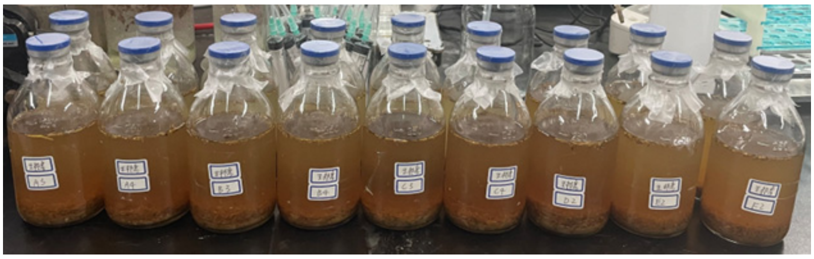

Figure S1. The experimental reactors.

References

[1] Zhang B., Li Y., Fei Y., Cheng Y. Novel pathway for vanadium (V) bio-detoxification by gram-positive Lactococcus raffinolactis. Environmental Science & Technology, 2021, 55(3): 2121-2131.

[2] Wang G, Zhang B, Li S, Yang M., Yin C. Simultaneous microbial reduction of vanadium (V) and chromium (VI) by *Shewanella loihica* PV-4. Bioresource Technology, 2017, 227: 353-358.

[3] Shi C., Cui Y., Lu J., Zhang B. Sulfur-based autotrophic biosystem for efficient vanadium (V) and chromium (VI) reductions in groundwater. Chemical Engineering Journal, 2020, 395: 124972.

[4] Ali M.M.S., Imam D.M., El-Nadi Y.A. Vanadium (V) removal and recovery by adsorption onto modified activated carbon derived from natural hydroxyapatite. Journal of the Iranian Chemical Society, 2021, 18(10): 2771-2784.

[5] He Q., Si S., Zhao J., Yan H., Sun B., Cai Q., Yu Y. Removal of vanadium from vanadium-containing wastewater by amino modified municipal sludge derived ceramic. Saudi Journal of Biological Sciences, 2018, 25(8): 1664-1669.
